# Simple Technique for Removing Broken Pedicular Screws

**DOI:** 10.5704/MOJ.1403.003

**Published:** 2014-03

**Authors:** A Agrawal

**Affiliations:** Department of Neurosurgery, Narayana Medical College Hospital, Nellore, India

## Abstract

The procedure for removing a broken pedicle screw should ideally be technically easy and minimally invasive, as any damage to the pedicle, during removal of the broken screw, may weaken the pedicle, thus compromising on the success of re-instrumentation. We describe the case of a 32-year old man who had undergone surgery for traumatic third lumbar vertebral body fracture three years prior to current admission and had developed the complication of pedicle screw breakage within the vertebral body. The patient underwent re-exploration and removal of the distal screws. Through a paravertebral incision and muscle separation, the screws and rods were exposed and the implants were removed.

**Key Words:**

Implant failure, spine surgery, pedicle screws, spinal fracture, spinal instrumentation

## Introduction

It has been recognized that pedicle screw fixation in spinal surgery produces a higher rate of fusion compared with other methods^1-6^. At the same time it may be associated with a higher rate of complications and so the safety and the effectiveness of pedicle-screw instrumentation have been questioned ^3, 6^. We describe a rare complication of pedicle screw breakage within the vertebral body and discus the management including the technique of screw removal.

## CASE REPORT

**Fig. 1: X-ray dorso lumbar spine AP and lateral view showing broken hardware.**
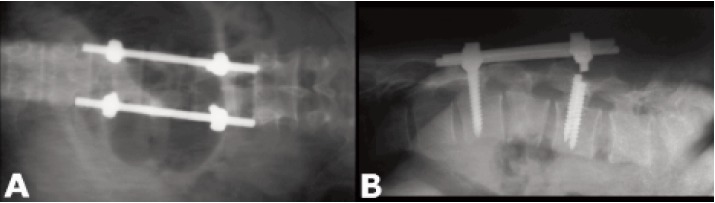


**Fig. 2: Clinical photograph showing protrusion of the screw at lower end more on left side.**
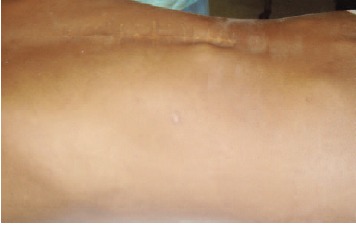


**Fig. 3: Clinical photograph showing screws were replaced with larger diameter screws.**
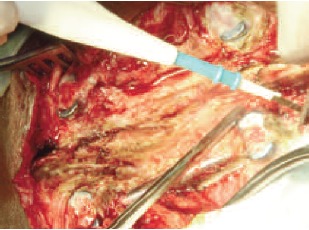


A 32-year old man had undergone pedicle screw surgery for -third lumbar vertebral body fracture three years previously. Following surgery, he had been apparently well, until six months prior to the current presentation, he had complained of local pain at the site of surgery which increased with stress and work. Bowel and bladder functions were normal. Motor power and sensation in the lower limbs were normal. Lumbar spine x-ray showed breakage of both distal screws at the neck of the screws (Figure 1). The patient was planned for exploration and removal of the distal screws. Through paravertebral dissection and muscle separation, the screws and rods were exposed and the rods accompanying the pedicle screws were removed. The proximal parts of the broken screw were removed (Figure 2)and (Figure 3). The retained deep fragments were within the surface of the bone in the pedicle (Figure 3). Compact bone which had partially embedded the threads and - the ends of the fragments was removed. As a first step a groove was created around the impacted part of the screw and a minimum of two grooves in the screw were exposed. The screws were held tightly with plier and gradually threaded out of the bone. During surgery both the distal (caudal) screws were removed (Figure 3) and replaced with larger diameter screws through the same entry points. The position was confirmed with image intensification. The patient was well with complete relief of pain at one year follow up.

## Discussion

Pedicle screw breakage is reported to occur in 1-11.2% of inserted screws and in 0.4-24.5% of patients^3, 5^. This implant failure can be a result of pseudarthrosis and can lead to pedicle screw or rod breakage^5^. By using the finite element model Chen et al demonstrated that screws on the caudal end were subjected to larger axial stress than those on the cephalic end, supporting the clinical finding that 75% of patients had screw breakage caudally (as in present case. In addition, screws show fatigue striations and ductile fracture around the edge^1^. Appropriate radiographs can demonstrate the screw breakage and revised spinal surgery is the mainstay of treatment when there is a broken pedicle screw^1^. Removal of the broken screw can be a difficult task and many techniques have been described to remove deeply embedded broken pedicle screw fragments^1, 3^. The methods described to remove broken screws include creating a deep pilot hole in the center of the fractured screw and engaging the screw driver to reverse and rotate the screw counterclockwise, whereby the retained screw fragment is backed out of the hole, preserving the bone interface^6^. Alternatively, using a high-speed drill with a long bit - to make a slot in the top surface of the broken screw to accommodate a standard flathead screwdriver to remove the screw3. Both of these techniques have been used successfully to extract broken pedicle screws, while maintaining the integrity of the pedicle^3^. These procedures can be limited by the availability of drill and other equipment and also deep drilling inside the broken screw for the application of screw extractor is usually difficult and the additional effort in tacking of the screw extractor towards the broken screw carries the risk of moving the broken screw deeper^4^. In another method, wide bone removal of the pedicle at the periphery of the broken screw can help to grasp the broken screw by retrieval instruments and remove the screw by counter clockwise movement with the help of friction and grasping, respectively^2^. These methods may necessitate the removal of a large amount of bone around the broken screw tip for application of clamp, and clamp failure during retrieving can be a major problem^4^. After successful removal of the broken screws larger diameter screws can be replaced^4^.

**Fig. 4: Broken screws and screw heads.**
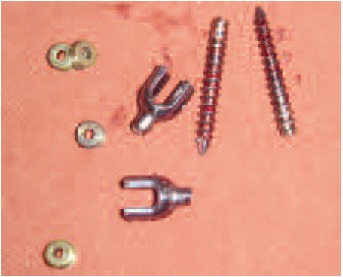


## Conclusion

It has been suggested that many complications can be prevented by careful attention to the application of established surgical techniques, an understanding of the dynamic stabilization system, and proper selection of patients. The removal process of a broken embedded pedicle screw should be technically easy and noninvasive to the pedicle, as any damage to the pedicle, during removal of the broken screw, may weaken the pedicle, thus compromising on the success of re-instrumentation.
